# A Physiologically Based Pharmacokinetic Model for Predicting Diazepam Pharmacokinetics after Intravenous, Oral, Intranasal, and Rectal Applications

**DOI:** 10.3390/pharmaceutics13091480

**Published:** 2021-09-15

**Authors:** Sundus Khalid, Muhammad Fawad Rasool, Imran Imran, Abdul Majeed, Hamid Saeed, Anees ur Rehman, Waseem Ashraf, Tanveer Ahmad, Yousef A. Bin Jardan, Faleh Alqahtani

**Affiliations:** 1 Department of Pharmacy Practice, Faculty of Pharmacy, Bahauddin Zakariya University, Multan 60800, Pakistan; sunduskhalid.sk@gmail.com (S.K.); abdulmajeed@bzu.edu.pk (A.M.); aneesurrehman@bzu.edu.pk (A.u.R.); 2 Department of Pharmacology, Faculty of Pharmacy, Bahauddin Zakariya University, Multan 60800, Pakistan; imran.ch@bzu.edu.pk (I.I.); chishtiwaseem@yahoo.com (W.A.); 3 Section of Pharmaceutics, University College of Pharmacy, Allama Iqbal Campus, University of the Punjab, Lahore 54000, Pakistan; hamid.pharmacy@pu.edu.pk; 4 Institute for Advanced Biosciences (IAB), Centre National de la Recherche Scientifique, Unite Mixte de Recherche 5309, Institut National de la Sante et de la Recherche Medicale U1209, Grenoble Alpes University, 38700 La Tronche, France; tanveer.ahmad@univ-grenoble-alpes.fr; 5 Department of Pharmaceutics, College of Pharmacy, King Saud University, Riyadh 11451, Saudi Arabia; ybinjardan@ksu.edu.sa; 6 Department of Pharmacology and Toxicology, College of Pharmacy, King Saud University, Riyadh 11451, Saudi Arabia

**Keywords:** physiologically based pharmacokinetic (PBPK), Simcyp^®^, diazepam, intranasal, rectal, route of administration, pharmacokinetics

## Abstract

Diazepam is one of the most prescribed anxiolytic and anticonvulsant that is administered through intravenous (IV), oral, intramuscular, intranasal, and rectal routes. To facilitate the clinical use of diazepam, there is a need to develop formulations that are convenient to administer in ambulatory settings. The present study aimed to develop and evaluate a physiologically based pharmacokinetic (PBPK) model for diazepam that is capable of predicting its pharmacokinetics (PK) after IV, oral, intranasal, and rectal applications using a whole-body population-based PBPK simulator, Simcyp^®^. The model evaluation was carried out using visual predictive checks, observed/predicted ratios (R_obs/pred_), and the average fold error (AFE) of PK parameters. The Diazepam PBPK model successfully predicted diazepam PK in an adult population after doses were administered through IV, oral, intranasal, and rectal routes, as the R_obs/pred_ of all PK parameters were within a two-fold error range. The developed model can be used for the development and optimization of novel diazepam dosage forms, and it can be extended to simulate drug response in situations where no clinical data are available (healthy and disease).

## 1. Introduction

The amnesic and anxiolytic properties of benzodiazepines make this pharmacological class clinically useful in several indications, including insomnia, anxiety, muscle relaxation, and epilepsy, but their use is also associated with several unwanted side effects [1,2]. Among the benzodiazepines, diazepam is the most commonly prescribed medium potency long-acting drug [3,4]. Diazepam is uniquely metabolized in the liver into a pharmacologically active metabolite, *N*-desmethyldiazepam, by demethylation and hydroxylation [2,5,6]. Diazepam shows wide interindividual variability in its metabolism, which results in marked differences in its systemic concentrations. Moreover, the genetic variability in the expressions of CYP2C19 and CYP3A4 can affect its therapeutic efficacy and may lead to adverse drug reactions [7,8,9,10].

Diazepam is commonly employed as an anxiolytic and anticonvulsant in intravenous (IV), oral, intramuscular, intranasal, and rectal dosage forms [3,4]. It is administered through different routes of drug administration for the management of acute and chronic diseases [11]. Parenteral diazepam is preferred in hospitals because of its rapid action (especially to suppress seizures), but this route of administration is not convenient in ambulatory settings and is also associated with serious side effects, i.e., hypotension, dysrhythmias, etc. [12,13]. The other routes of administration, i.e., oral, intranasal, and rectal are considered more valuable when the IV route becomes inconvenient. Diazepam’s high permeability leads to rapid drug absorption via the rectal route, and diazepam is the only approved drug by U.S Food and Drug Administration (FDA) for rectal administration for out-patient treatment of early status epilepticus specifically in pediatrics [14,15,16]. On the other hand, the intranasal passage is an alternative to the oral and rectal route for diazepam administration for treating acute seizure management [17,18]. For any treatment, the therapeutic goal is only achieved if the drug is effective through the specified route of administration without causing any harm. To facilitate the clinical use of diazepam, there is a need to develop other non-oral formulations for humans when the oral route of administration is not convenient such as transdermal applications. Although such formulations have been examined and assessed in animals, they have not yet been tested in humans [19,20,21]. The development of non-oral dosage forms in humans can be facilitated by utilizing Physiologically Based Pharmacokinetics (PBPK), which presents countless opportunities for improvement in drug development in healthy and diseased populations [22].

PBPK models incorporate drug-dependent parameters along with the population-dependent system parameters in the presence of intrinsic or extrinsic factors to estimate the pharmacokinetics (PK) of the drug [23]. The concept of PBPK was established in 1937 [24], almost a decade ago, but for the last few years, PBPK modeling has been used as a tool for drug development and discovery, as described in several literature reviews [25,26,27,28]. PBPK models not only predict the clinical PK of a chemical entity but also reveal variables that may influence drug development, i.e., drug–drug interactions (DDI) or disease-states [29,30]. By allowing for the incorporation of in vitro drug release data, the PBPK modeling platforms [31,32] can be used to estimate the absorption, distribution, metabolism, and excretion (ADME) of the drug, which can assist in designing and optimizing novel dosage forms [33].

There are few published reports for the PBPK models for diazepam, which are focused on predicting its PK and DDIs in both humans and animals [34,35,36]. One study reported a PBPK model for diazepam to evaluate its DDIs with opioids (oxycodone, buprenorphine, and fentanyl) [35]. In contrast, another study aimed to develop a diazepam PBPK model by using different modeling and simulation tools after IV application only [34]. The third reported diazepam PBPK model was focused on the prediction of drug parameters in rats [36]. Since diazepam is administered through different routes of administration (IV, oral, intranasal, and rectal), if a PBPK model that can predict its PK after application of different dosage forms is developed, it may have many clinical applications. Keeping this in mind, a PBPK diazepam model was developed using a systematic model-building approach [37,38] that was capable of predicting its ADME through different routes of drug administration. This study aims to develop a PBPK model in a healthy population for the prediction and evaluation of diazepam pharmacokinetics after the administration of the drug through different routes, i.e., IV, oral, intranasal, and rectal.

## 2. Methods

### 2.1. Modeling Software

Simcyp^®^ version 19 release 1 (Simcyp Ltd., Sheffield, UK), a population-based simulator, was employed for diazepam PBPK model development.

### 2.2. Modeling Approach

**Figure 1:**
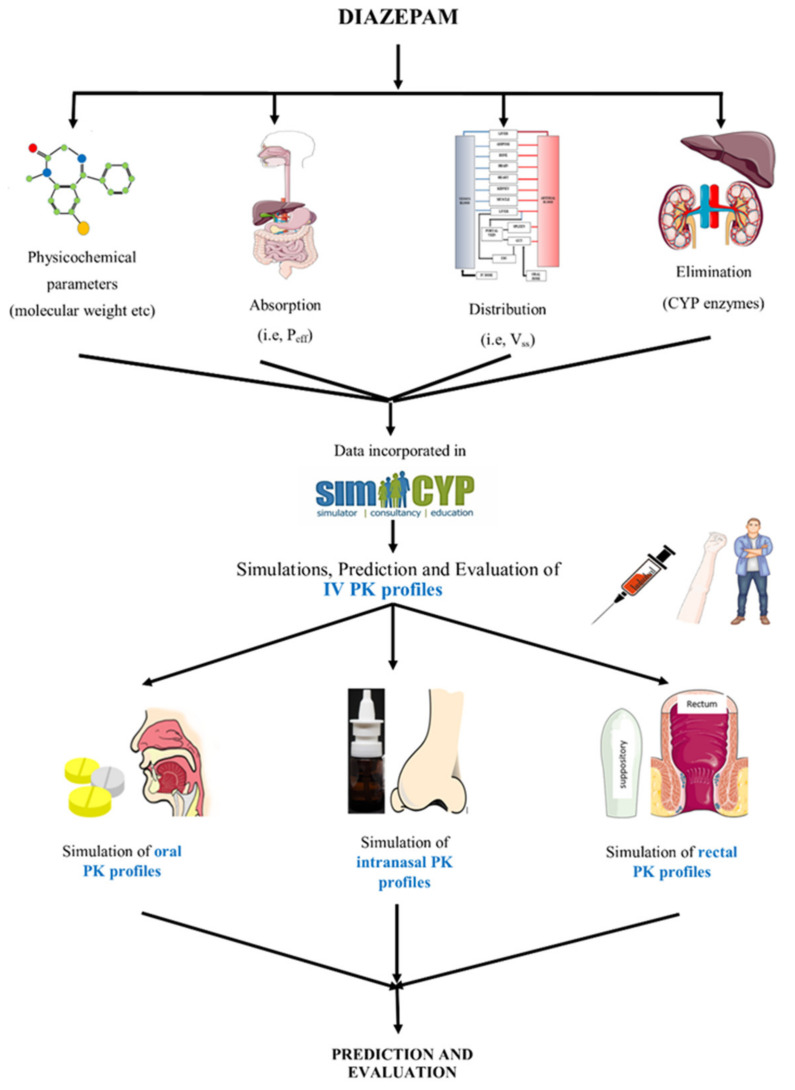
Systematic diagram for the development of the PBPK model of diazepam. Human jejunum permeability (P_eff_), the volume of distribution at steady-state (V_ss_), pharmacokinetic (PK), and intravenous (IV). The figure was produced using “Servier Medical Art” (https://www.smart.servier.com, accessed on 12 September 2021).

The process of PBPK model development commenced with the literature search for the extraction of drug-specific and population-specific parameters. The obtained data, i.e., molecular weight, fraction absorbed (f_a_), blood to plasma ratio (B:P), etc. were incorporated into the program to simulate and evaluate the IV profiles in the healthy adults. After the IV model was successfully developed in the adult population, oral PK profiles were simulated by incorporating oral drug absorption parameters, i.e., human jejunum permeability (P_eff_), etc. After successful oral data assessment, the clinical PK profiles of other routes of administration for diazepam were evaluated, i.e., intranasal and rectal. The layout for the developed diazepam PBPK model is given in Figure 1.

### 2.3. Model Parameters

The parameterization of the model began with a thorough review of the published literature, including in vitro and in vivo data for diazepam. The physicochemical properties of diazepam including molecular weight, octanol–water partition coefficient (LogP_o:w_), and acid dissociation constant (pKa) are given in Table 1. For estimating drug absorption via oral, intranasal, and rectal routes, the advanced dissolution, absorption, and metabolism (ADAM) model was utilized. The P_eff_ was predicted using the number of hydrogen bond donors (HBD) and polar surface area (PSA). The predicted P_eff_ value for diazepam was 12.434 × 10^−4^ cm/s by incorporating PSA and HBD values of 32.67 A^o^ and 0, respectively [39]. For the prediction of diazepam distribution in an adult population, a whole-body full PBPK model was applied for estimating the tissue-to-plasma partition co-efficient and volume of distribution at steady-state (V_ss_) using “Method-3” (The Rodger and Rowland method + ion membrane permeability) within the Simcyp^®^. The Michaelis-Menten constant (K_m_) and maximum rate of reaction (*V_max_*) for CYP2B6, CYP2C19, CYP3A4, and CYP3A5 were used for predicting drug clearance. The final diazepam-specific input parameters for the developed PBPK model are given in Table 1.

### 2.4. Pharmacokinetic Data

A detailed online literature search was executed to categorize the PK profiles (plasma concentration vs. time profiles or data) of diazepam in the adult population for model development. The studies were selected based on the PK profiles, the route of administration (IV, oral, intranasal, and rectal), the dose administered, age, the female proportion, and the duration of the study. The observed data were extracted by scanning PK profiles through GetData Graph Digitizer V.2.26.0 (http://getdata-graph-digitizer.com, accessed on 12 September 2021) [45].

In conclusion, 8 clinical studies and 20 concentrations vs. time profiles were added for diazepam model development; out of these 20 profiles, 6 were based on IV administration (71 individuals), 5 were profiles of oral (88 individuals), 6 were of intranasal administration (66 individuals), and 3 were of rectal diazepam administration (38 individuals) were used. Among all of these PK profiles, about one-third (two IV, two oral, two intranasal, and one rectal) were employed for diazepam PBPK model development and the remaining two-third (four IV, three oral, four intranasal, and two rectal) were utilized for verification of the model. For model evaluation, all of the observed clinical PK data were used. Table 2 shows the demographic data utilized for diazepam model construction and evaluation in the adult populations.

### 2.5. Model Evaluation

For every PK profile, 100 individuals were selected as a virtual population to perform a simulation by incorporating the parameters, i.e., the administration route, age range, female proportion, dosing, and study duration, as mentioned in the above-published studies. The evaluation of the developed diazepam PBPK model was initially performed with visual predictive checks by overlaying mean observed PK profiles on predicted data, which include the mean, 5th percentile and 95th percentile, and maximum and minimum predicted values. A non-compartmental analysis (NCA) was performed for the comparison of observed and predicted values of PK parameters including clearance (CL), area under the plasma concentration vs. time curve (AUC_0–t_), and maximum plasma concentration (C_max_) values. Moreover, the fold-error (observed/predicted ratios) and average fold error (AFE) of AUC_0–t_, C_max,_ and CL were calculated using Equations (1) and (2). A two-fold error range (within 0.5−2-fold range) was used for the evaluating ratios (R_obs/pred_) for PK parameters [53].

Mean observed/predicted ratio

(1) $${\mathrm{ratio}}_{\left(\frac{\mathrm{obs}}{\mathrm{pred}}\right)}=\frac{\mathrm{observed}\;\mathrm{value}\;\mathrm{of}\;\mathrm{PK}\;\mathrm{parameter}}{\mathrm{predicted}\;\mathrm{value}\;\mathrm{of}\;\mathrm{PK}\;\mathrm{parameter}}$$

Average fold error

(2) $$\mathrm{AFE}=10^{\frac{\sum \log\left(\mathrm{fold}\;\mathrm{error}\right)}{N}}$$

Additionally, the values of bioavailability parameters such as F_a_ (fraction of oral dose absorbed from the intestinal lumen), F_g_ (fraction of drug that escaped both intestinal first-pass metabolism and transporter-secretion available at hepatic portal blood), F_h_ (fraction of drug escaping hepatic first-pass elimination) were predicted after simulating oral and intranasal profiles. Through these parameters, the oral and intranasal data can be evaluated using predicted bioavailability, which can be calculated using the following equation:

(3) $$F=F_{a}\cdot F_{g}\cdot F_{h}$$

## 3. Results

### 3.1. Intravenous Dose Administration in the Adult Population

**Figure 2:**
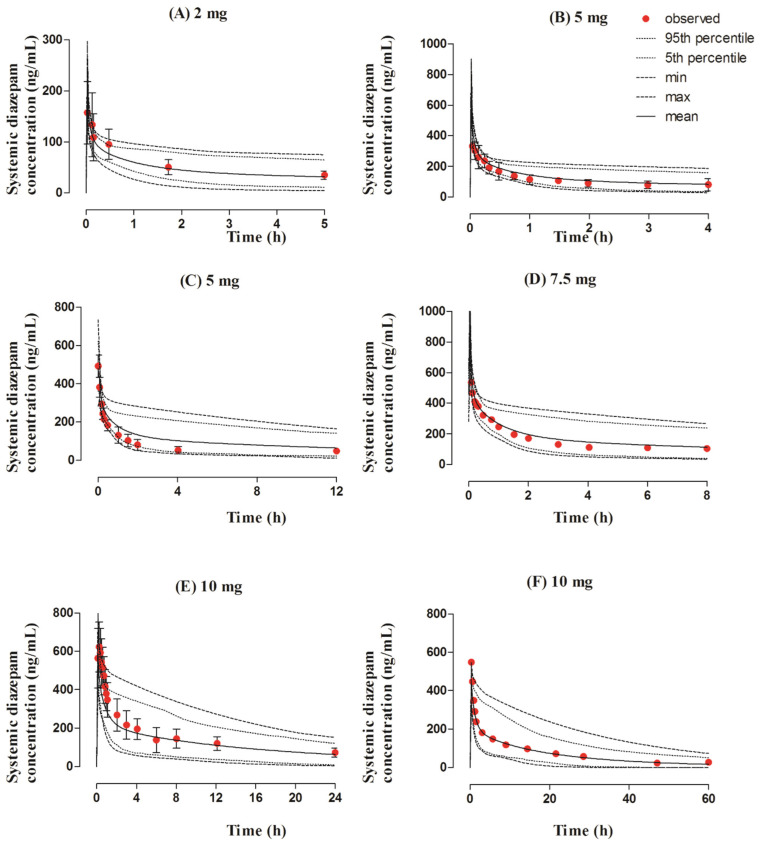
Observed and predicted systemic concentration profiles of diazepam after IV application. The solid line (―) represents the mean value; the dotted lines (….) indicate the 5th and 95th percentiles; the dash line (---) shows the maximum and minimum predicted values; and the red filled circles (•) represent the mean observed data along with the standard deviation, where available. The figure contains systemic diazepam concentration profiles after administering IV doses of (**A**) 2 mg [46], (**B**) 5 mg [47], (**C**) 5 mg [48], (**D**) 7.5 mg [49], (**E**) 10 mg [15], and (**F**) 10 mg [50].

**Figure 3:**
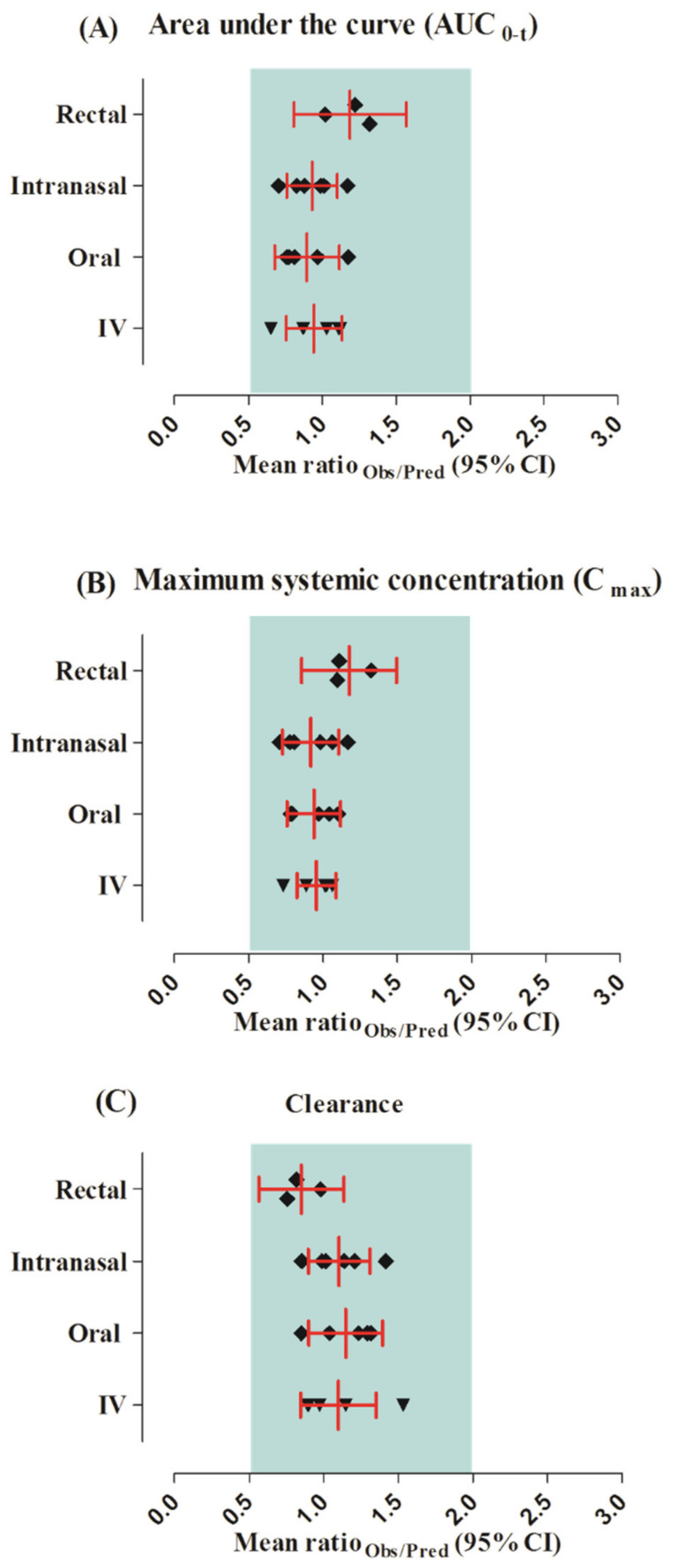
Mean ratio observed/predicted for the pharmacokinetic parameters of diazepam along with their 95% confidence intervals after IV, oral, intranasal, and rectal application. (**A**) Area under the curve (AUC_0-1_), (**B**) maximum systemic concentration (C_max_), (**C**) clearance.

The observed and predicted systemic diazepam concentration profiles can be seen in Figure 2. The visual predictive checks showed that the developed model successfully predicted diazepam PK after its IV administration within the dose range of 2–10 mg. The mean AUC_0–t_ and C_max_ R_obs/pred_ were 0.94 (95% CI 0.75−1.13) and 0.95 (95% CI 0.82−1.08), respectively. All of the PK parameters were within the two-fold error range (Table 3 and Table 4, Figure 3).

### 3.2. Oral Dose Administration in the Adult Population

**Figure 4:**
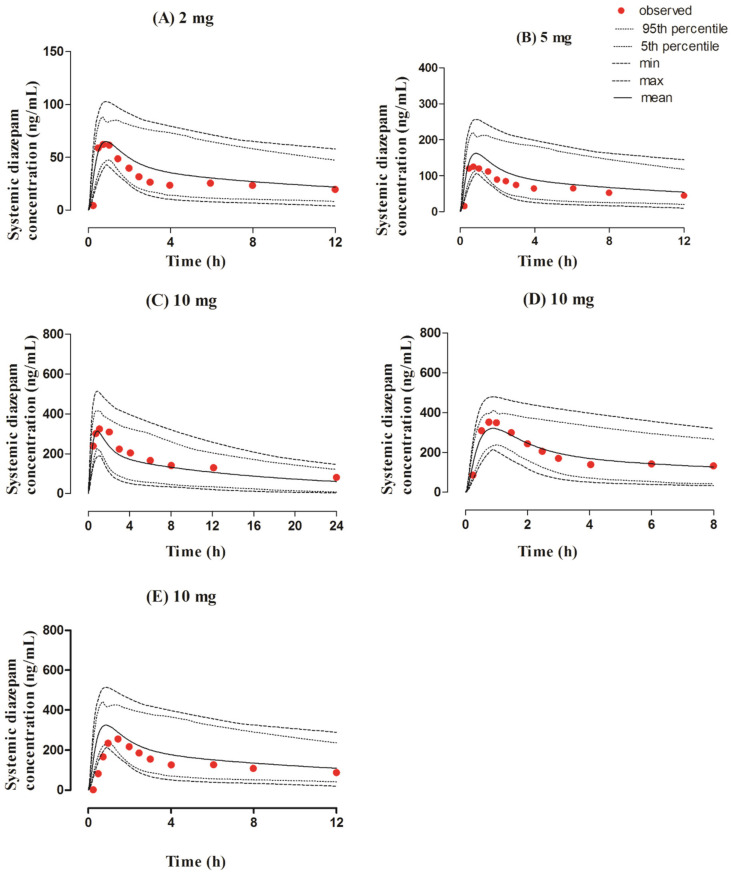
Observed and predicted systemic concentration profiles of diazepam after oral application. The solid line (―) represents the mean value, the dotted lines (….) indicate the 5th and 95th percentiles, the dash line (---) shows the maximum and minimum predicted values, and the red filled circles (•) represent the mean observed data. The figure contains systemic diazepam concentration profiles after administering oral doses of (**A**) 2 mg [51], (**B**) 5 mg [51], (**C**) 10 mg [15], (**D**) 10 mg [52], and (**E**) 10 mg [51].

The observed and predicted concentration profiles after the oral administration of a 2–10 mg dose of diazepam are given in Figure 4. The observed clinical data was in between the maximum and minimum values, as perceived by visual predictive checks. The mean R_obs/pred_ of C_max_ and CL were within the range of the two-fold error, as shown by the values of 0.93 (95% CI 0.75–1.11) and 1.14 (95% CI 0.90−1.39), respectively (Table 3 and Table 4, and Figure 3).

### 3.3. Intranasal Dose Administered in the Adult Population

**Figure 5:**
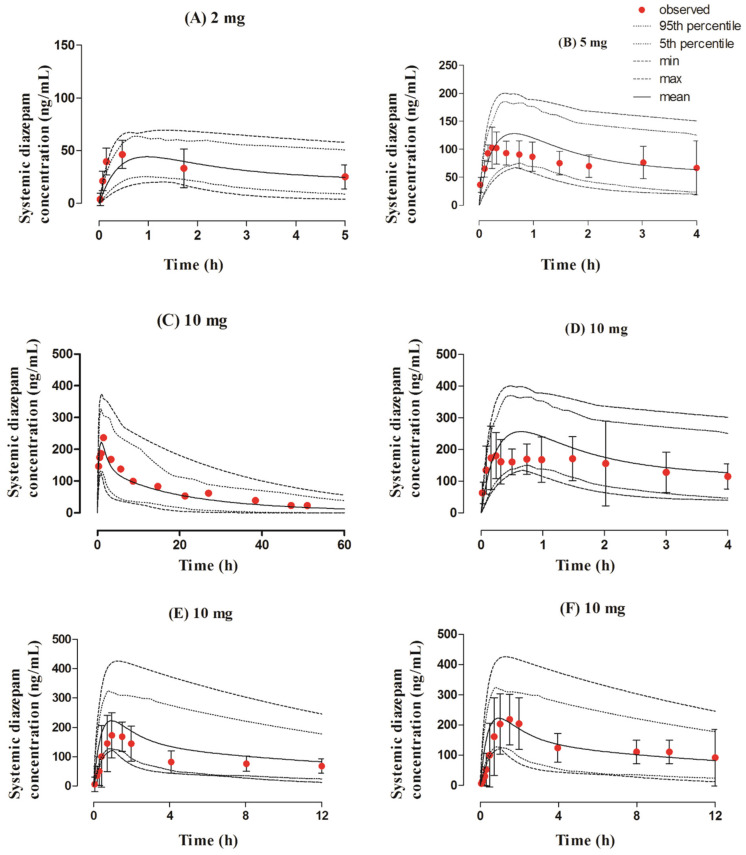
Observed and predicted systemic concentration profiles of diazepam after intranasal application. The solid line (―) represents the mean value; the dotted lines (….) indicate the 5th and 95th percentiles; the dash line (---) shows the maximum and minimum predicted values; and the red filled circles (•) represent the mean observed data along with the standard deviation, where available. The figure contains systemic diazepam concentration profiles after administering intranasal doses of (**A**) 2 mg [46], (**B**) 5 mg [47], (**C**) 10 mg [50], (**D**) 10 mg [47], (**E**) 10 mg [48], and (**F**) 10 mg [48].

The developed model effectively predicts diazepam concentration–time profiles after administration of the intranasal dose of 2–10 mg in adult individuals, as shown in Figure 5. The visual predictive checks represented that these predictions were in accordance with the observed data. Additionally, the PK parameters depicted the values within a two-fold range (0.5–2.0); the mean R_obs/pred_ of AUC_0–t_ was 0.93 (95% CI 0.76–1.10) and the C_max_ value was 0.91 (95% CI 0.72–1.10), which can be seen in Table 3 and Table 4, and Figure 3.

### 3.4. Rectal Dose Administered in the Adult Population

**Figure 6:**
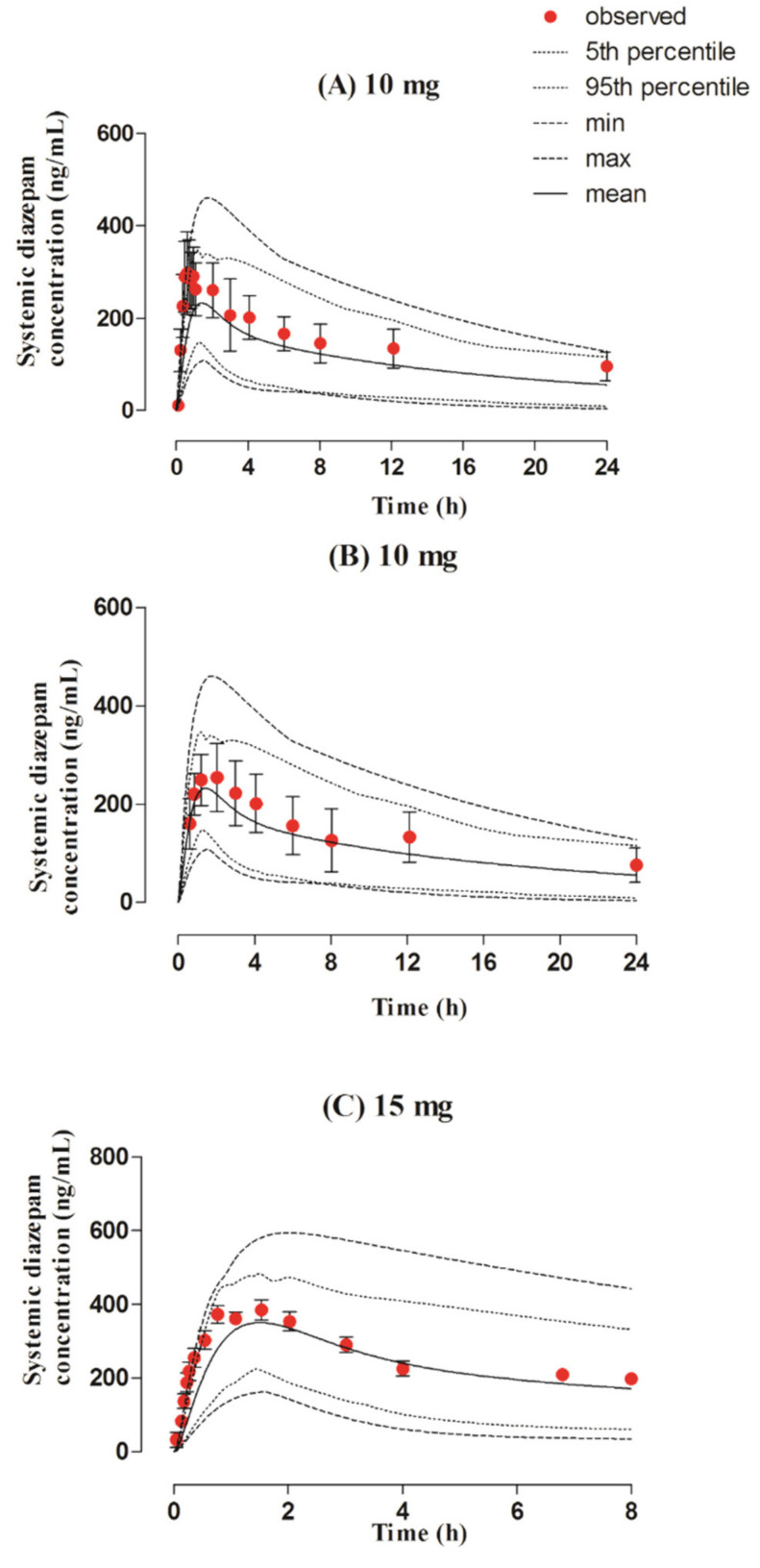
Observed and predicted systemic concentration profiles of diazepam after rectal application. The solid line (―) represents the mean value; the dotted lines (….) indicate the 5th and 95th percentiles; the dash line (---) shows the maximum and minimum predicted values; and the red filled circles (•) represent the mean observed data along with the standard deviation, where available. The figure contains systemic diazepam concentration profiles after administering rectal doses of (**A**) 10 mg [15] (**B**) 10 mg [15], and (**C**) 15 mg [49].

The predicted diazepam concentration–time profiles after rectal administration doses of 10–15 mg were in complete agreement with the observed PK data (Figure 6). These results were further confirmed by the mean R_obs/pred_ of the PK parameters of diazepam being within the two-fold error range (Table 3 and Table 4, and Figure 3) as AUC_0–t_ after the rectal application was 1.18 (95% CI 0.80–1.56). Additionally, the AFE values represented that the model effectively predicted PK parameters after rectal administration.

## 4. Discussion

In the presented work, a PBPK model was developed to predict the ADME of diazepam after IV, oral, intranasal, and rectal administration. The systematic model building was commenced by predicting IV clinical profiles in adults by incorporating all drug and population-specific parameters in Simcyp^®^. After successful evaluation of IV profiles, absorption parameters were incorporated for the prediction and evaluation of oral PK clinical data. Intranasal and rectal PK profiles were also evaluated in the same way as IV and oral data.

The developed diazepam PBPK model represented comparable drug disposition in adults after IV administration as the mean R_obs/pred_ value of AUC_0–t_ with 95% CI was 0.94 (95% CI 0.75–1.13). Although diazepam is used as a first-line agent in the emergency management of seizures, it is difficult to administer IV diazepam to stop seizures in an ambulatory setup. The suitable diazepam alternatives in the outpatient setting are non-IV formulations.

The presented model successfully simulated oral diazepam PK data, and the observed and predicted systemic concentrations were in close association with the reported data as the mean R_obs/pred_ C_max_ value with 95% CI was 0.93 (95% CI 0.75–1.11). Moreover, the predicted bioavailability after the oral application was in the range of 76–97%, which is comparable with the reported value of 94% [54]. The oral route of administration is not feasible in suppressing seizures due to its late onset of action and congestion problems. Due to this reason, clinicians prefer other routes of administration, i.e., rectal and intranasal, for diazepam administration to suppress seizures using rapid diazepam action.

Apart from the IV and oral routes, the intranasal route of administration was used as an alternative for drug administration from the early 1980s [55]. The intranasal route is commonly employed because of its accessibility, its rapid action without first-pass metabolism, its patient conformity, and its noninvasiveness [56,57,58]. Diazepam is considered effective for intranasal administration due to its physicochemical properties and provides extended action compared with other benzodiazepines [59]. This intranasal route for diazepam is more attractive for the user compared with some other formulations, i.e., rectal [11]. For intranasal PK profiles of diazepam, the abovementioned strategy of IV and oral formulations were implemented with additional parameters, i.e., f_a_ and k_a_. The presented model includes multiple intranasal formulations, i.e., solution, suspension, and supersaturated solutions. The supersaturated solution of diazepam is expected to be rapidly absorbed across the nasal mucosa soon after administration, which leads to the shorter residence time of the drug at the absorption site along with nasal drainage, which contributes to a decrease amount of drug available for absorption over time [60,61]. Therefore, in the case of a supersaturated solution, compared with other intranasal formulations, k_a_ changed to accommodate the increased drug absorption because of rapid permeation in a short time. The model effectively predicted the overall PK parameters as R_obs/pred_ with a 95% CI of AUC_0–t_ was calculated as 0.93 (95% CI 0.76–1.10). Thus, a solution with a drug given through the intranasal route is absorbed more rapidly than suspension [62], but the developed model showed no significant increase in drug disposition as the AUC_0–t_ of the intranasal solution (1532 ng.h/mL) was slightly higher than that of the intranasal suspension (1523 ng.h/mL). Furthermore, the reported bioavailability after intranasal administration was 97% [48,63], and the model predictions were in the range of 81–97%.

The other non-oral route for diazepam administration that has rapid absorption and showed up to 80% to 90% bioavailability is the rectal route. The therapeutic concentration of diazepam after rectal administration was achieved within 5 to 10 min, similar to that of IV administration. However, the use of a rectal formulation has social limitations in terms of patient conformity [16,64,65]. In the recent past, a diazepam gel for rectal administration was the only drug available with an immediate action for suppressing seizures in ambulatory settings [66]. The presented study, successfully predicted the PK profiles of different rectal formulations, i.e., suppository, solution, and gel, that can be recognized with comparable values of PK parameters. The mean R_obs/pred_ with a 95% CI of CL was calculated as 0.85 (95% CI 0.56–1.13). The predicted AUC_0-8_ of the rectal gel (2028.11 ng.h/mL) [49] was lower than that of the AUC_0-24_ of the suppositories (2666.01 ng.h/mL) [15]. Although IV diazepam is commonly used to suppress prolonged seizures, seizures need to be stopped immediately at home before hospitalization. Apart from the intranasal route of diazepam, its rectal dosage forms are commonly employed to control seizures, as the only FDA-approved diazepam rectal gel (Diastat^®^, Bausch Health NJ, USA) is intended for use in pediatrics [67]. The developed PBPK model may be used to optimize the other non-oral formulations (transdermal) in humans by utilizing the in vitro data (dissolution data) of the drug using the already reported strategies [68].

Ideally, the treatment option should be chosen based on disease type, rapid onset of action, easy administration, and extended duration with minimal adverse effects [11]. In terms of route of administration, diazepam has various pros and cons, where IV administration has a swift onset of action, but at the same time, it is not convenient in the ambulatory setting, and on the other hand, the oral route has slow absorption and administration issues, i.e., swallowing in seizure [16]. Intranasal and rectal routes of administration also have unwanted effects, i.e., irritation at the site of administration, but they are commonly employed for treating seizures because of their rapid action and minimal side effects [11]. The comparative evaluation of the intranasal and the rectal formulations suggests that intranasal dosage forms are associated with a high variability in drug exposure and they often require the optimization of spray devices [60].

The previously published PBPK models for diazepam were focused on predicting its PK and DDIs in both humans and animals [34,35,36]. One study has used two Bayesian software for the development of a PBPK model of diazepam after the administration of an IV infusion only. Both tools produced very good fits at the individual and population levels [34]. The other study reported the development of full and minimal PBPK models for the evaluation of DDIs between opioids (fentanyl, oxycodone, and buprenorphine) and benzodiazepines (alprazolam, diazepam, midazolam, and triazolam). Full PBPK models were applied to diazepam and all opioids, while minimal PBPK models were developed for all benzodiazepines except diazepam [35]. The third reported PBPK model accounted for the parameter variability and uncertainty in the presence of qualitative and semi-quantitative data. The two approaches for the incorporation of parameter variability and uncertainty used in the study included the fuzzy and Monte Carlo simulations [36]. However, to date, there is no report for a PBPK model for diazepam that has been developed and evaluated in the adult population after implementing a systematic model-building approach. This is the first report of a whole-body full PBPK model for diazepam that has successfully predicted its PK after IV, oral, intranasal, and rectal administration.

## 5. Conclusions

Our diazepam PBPK model successfully predicted diazepam PK in the adult population after doses administered through IV, oral, intranasal, and rectal routes. The non-oral routes for diazepam administration are preferred in an ambulatory setting, especially as the IV route in seizing patients is not convenient in an outpatient setting and as it may lead to adverse effects. The developed model can be used for the development and optimization of novel diazepam dosage forms, and it can be extended to simulate drug response in situations where no clinical data are available as in diseased states and special populations (pediatrics).

## Figures and Tables

**Table 1:** Drug-specific input parameters of diazepam.

Parameters	Values		Ref.
**Physicochemical Properties**			
Molecular Weight (g/mol)	284.74		[40]
Log*P*_o:w_	2.82		[35]
pKa	3.4		[41]
**Absorption**			
Model			
P_eff,man_ (cm/s)	12.434 × 10^−4 a,b^		[35]
PSA (A^o^)	32.67		[39]
HBD	0		[39]
**Distribution**			
**Model Prediction Method**	**The Rodger and Rowland Method + Ion Membrane Permeability**		
B/P ratio	0.58		[41]
f_u_	0.03		[3,42]
V_ss_ (L/kg)	0.66 ^a,c^		[35,43]
**For Intranasal Administration**			
Lung f_a_	0.7 ^d^		
Lung k_a_	1.6 ^d,e^		
**Elimination**			
f_umic_	0.59 ^a^		
**N-demethylation**	*V_max_* (pmol/min/pmol)	**K_m_** (μM)	
CYP2B6	3.6	113	[44]
CYP2C19	2.3	32	
CYP3A4	14.8	1828	
CYP3A5	1.8	293	
**3-hydroxylation**			
CYP2B6	0.1	150	[44]
CYP2C19	20.2	846	
CYP3A4	151.3	2235	
CYP3A5	48.4	316	

^a^ Value predicted by Simcyp, ^b^ reported value, ^c^ the reported value range was 0.59−1 L/kg, ^d^ adjusted by manual optimization, ^e^ adjusted to 3.2 while simulating supersaturated solution, blood-to-plasma ratio (B:P), fraction unbound (f_u_), Michaelis–Menten constant (K_m_), maximum rate of metabolic formation (*V_max_*), the volume of distribution at steady-state (V_ss_), human jejunum permeability (P_eff_), polar surface area (PSA), hydrogen bonding donor (HBD), acid dissociation constant (pKa), octanol–water partition coefficient (LogP_o:w_), fraction absorbed (f_a_), and absorption rate constant (k_a_).

**Table 2:** Population data for IV, oral, intranasal, and rectal administration of diazepam in the adult population.

No.	Population	No. of Subjects	Dose (mg)	Dosage Form	Age (Years)	Weight (kg)	Female Proportion	Ref.
**IV**								
1	Healthy	9	2	IV solution	20−30	58−80	0	[46]
2	Healthy	8	5	IV solution	Mean 28.3	-	0.33	[47]
3	Healthy	24	5	IV solution	18−45	Mean 71.8	0.2	[48]
4	Healthy	20	7.5	IV solution	Mean 28.8	Mean 72.6	0	[49]
5	Healthy	9	1	IV solution	18−25	58−70	0.33	[15]
6	Healthy	1	10	IV solution	26−37	60−85	0.2	[50]
**Oral**								
7	Healthy	11	2	-	19−35	-	0.27	[51]
8	Healthy	11	5	-	19−35	-	0.27	[51]
9	Healthy	48	10	Tablets	18−44	59.1−95	0	[52]
10	Healthy	11	10	-	19−35	-	0.27	[51]
11	Healthy	9	10	Tablet	18−25	58−70	0.33	[15]
**Intranasal**								
12	Healthy	9	2	Solution	20−30	58–80	0	[46]
13	Healthy	8	5	Supersaturated solution	Mean 28.3	-	0.33	[47]
14	Healthy	24	10	Solution	18−45	Mean 71.8	0.2	[48]
15	Healthy	24	10	Suspension	18−45	Mean 71.8	0.2	[48]
16	Healthy	8	10	Supersaturated solution	Mean 28.3	-	0.33	[47]
17	Healthy	1	10	Solution	26−37	60−85	0.2	[50]
**Rectal**								
18	Healthy	9	10	Solution	18−25	58−70	0.33	[15]
19	Healthy	9	10	Suppository	18−25	58−70	0.33	[15]
19	Healthy	20	15	Gel	Mean 28.8	Mean 173.9	0	[49]

**Table 3:** Observed and predicted values of PK parameters after IV, oral, intranasal, and rectal routes of administration.

	Dose (mg)	C_max_ (ng/mL)		Ratio	AUC_0–t_ (ng.h/mL)		Ratio	CL (L/h)		Ratio	Ref.
		Observed	Predicted		Observed	Predicted		Observed	Predicted		
**IV**											
1	2	157.06	154.39	1.01	285.45	256.60	1.11	7.00	7.79	0.89	[46]
2	5	332.28	452.92	0.73	455.11	522.20	0.87	10.98	9.57	1.14	[47]
3	5	492.1	479.77	1.02	851.74	1305.30	0.65	5.87	3.83	1.53	[48]
4	7.5	536.44	605.14	0.88	1244	1428.08	0.87	6.27	5.46	1.14	[49]
5	10	623	586.11	1.06	3505.37	3117.92	1.12	2.85	3.2	0.89	[15]
6	10	642.95	634.13	1.01	4491.90	4364.79	1.02	2.22	2.29	0.97	[50]
**Oral**											
1	2	62.07	63.97	0.97	330	406.55	0.81	6.06	4.91	1.23	[51]
2	5	125.24	159.95	0.78	784.19	1012.40	0.77	6.37	4.93	1.29	[51]
3	10	325	311.87	1.04	3487	2965.75	1.17	2.86	3.37	0.80	[15]
4	10	255.53	322.62	0.80	1540.28	2026.76	0.75	6.49	4.93	1.31	[51]
5	10	352	318.99	1.10	1445.96	1493.59	0.96	6.91	6.66	1.03	[52]
**Intranasal**											
1	2	46.25	39.66	1.16	161.73	159.94	1.01	12.36	12.5	0.98	[46]
2	5	102.66	127.34	0.80	309.82	352.62	0.87	16.13	14.18	1.13	[47]
3	10	218.15	222.08	0.98	1513.78	1532.92	0.98	6.60	6.52	1.01	[48]
4	10	172.99	222.48	0.77	1084	1523.59	0.70	9.22	6.56	1.41	[48]
5	10	180.51	254.69	0.70	588.64	710.80	0.82	16.98	14.06	1.20	[47]
6	10	236.82	222.70	1.06	3558.44	3033.84	1.17	2.81	3.29	0.85	[50]
**Rectal**											
1	10	297	224.17	1.32	3566.35	2699.95	1.32	2.80	3.70	0.75	[15]
2	10	254	229.40	1.10	3258.12	2666.01	1.22	3.06	3.75	0.81	[15]
3	15	384.86	350.74	1.09	2070.02	2028.11	1.02	7.24	7.39	0.97	[49]

**Table 4:** R_obs/pred_ and AFE values of PK parameters in an adult population after IV, oral, intranasal, and rectal administration of diazepam.

Parameters	R_obs/pred_	AFE
IV		
AUC_0–t_	0.94	0.93
CL	1.10	1.08
C_max_	0.96	0.95
Oral		
AUC_0–t_	0.90	0.89
CL	1.15	1.13
C_max_	0.94	0.93
Intranasal		
AUC_0–t_	0.93	0.92
CL	1.10	1.09
C_max_	0.92	0.90
Rectal		
AUC_0–t_	1.19	1.18
CL	0.85	0.85
C_max_	1.18	1.17
